# Quality-of-Life and Oncological Outcomes in Male Breast Cancer: Insights from an Extensive 20-Year Experience

**DOI:** 10.3390/cancers17050829

**Published:** 2025-02-27

**Authors:** Massimo Ferrucci, Francesco Milardi, Daniele Passeri, Maria Pozzerle, Matteo Cagol, Tania Saibene, Silvia Michieletto, Mariacristina Toffanin, Paola Del Bianco, Alberto Marchet

**Affiliations:** 1 Breast Surgery Unit, Veneto Institute of Oncology IOV, IRCCS-Istituto di Ricovero e Cura a Carattere Scientifico, 35128 Padova, Italy; 2 General Surgery, Department of Surgery, Oncology and Gastroenterology, University of Padua, 35121 Padova, Italy; 3 “Spalenza” Center-Don Gnocchi Foundation, 25038 Rovato, Italy; 4 Clinical Research Unit, Veneto Institute of Oncology IOV, IRCCS-Istituto di Ricovero e Cura a Carattere Scientifico, 35128 Padova, Italy

**Keywords:** male breast cancer, quality of life, patient satisfaction, breast cancer survival, breast cancer surgery

## Abstract

**Background/Objectives**: Male breast cancer (MBC) is a rare malignancy with a globally rising incidence. Due to the paucity of specific evidence, therapeutic strategies are often extrapolated from female breast cancer guidelines. The quality of life (QoL) among MBC patients remains underexplored. **Methods**: This single-institution retrospective study analyzed male patients who underwent surgery for newly diagnosed breast cancer at our institution from 1998 to 2020. The primary aim was to assess QoL after surgical and medical treatments using a newly developed 12-item dedicated questionnaire. Clinicopathological features were recorded, and oncological and survival outcomes were evaluated, with a specific focus on identifying factors influencing QoL. **Results**: A total of 109 consecutive MBC patients who underwent surgery were included, with 96.3% undergoing total mastectomy. The median global QoL score was 28.5 out of 36, where scores above 24 indicated a low impact of BC treatments on QoL. Multivariate analyses identified adjuvant chemotherapy (*p* < 0.001) and postoperative complications (*p* < 0.001) as significant predictors of poorer QoL. Patients reported significantly lower scores on medical treatment-related items compared to surgery-related items (*p* < 0.001). The 10-year overall survival rate was 79.1%, with a 10-year BC-specific mortality of 3.5%. Advanced age, higher stage disease, and receiving adjuvant chemotherapy were independently associated with poorer overall survival. **Conclusions**: Despite elevated mastectomy rates, MBC patients self-reported high levels of satisfaction with their treatment outcomes. Medical therapies showed a more pronounced negative impact on QoL than surgery. These findings provide novel insights into the QoL of MBC patients, highlighting the need for future prospective studies and tailored treatments.

## Simple Summary

Clinical evidence on male breast cancer (MBC) remains limited, and the quality of life (QoL) of MBC survivors is poorly understood. To address this gap, we conducted a retrospective study spanning 22 years, analyzing pathological, clinical, and survival data from male patients treated for breast cancer at a single institution. Using a novel and dedicated QoL questionnaire, we found that medical treatments have a more pronounced impact on QoL compared to surgical interventions. These insights provide a deeper understanding of the unique experiences and needs of MBC patients, highlighting the necessity for tailored treatment strategies and the development of validated QoL assessment tools specifically designed for male patients.

## 1. Introduction

Male breast cancer (MBC) is a rare condition, representing less than 1% of all breast malignancies and less than 1% of tumors diagnosed in men [1]. The lifetime risk of MBC is consistently lower than that of female breast cancer (FBC), with an estimated incidence of approximately 1 in 1000, compared to about 1 in 8 for women [1]. Nonetheless, the incidence of MBC is rising worldwide [2], with reported increases of 7.2% to 10.3% over the past decade [3].

Men represent less than 0.01% of the total study population across 131 randomized clinical trials on BC [4]. Consequently, most available data are derived from small retrospective studies, which lack the robustness needed for developing specific management recommendations. As a result, treatment approaches for MBC are frequently based on guidelines for female patients despite distinct clinical characteristics [5]. Diagnosis is frequently clinical and delayed, largely due to the absence of targeted screening programs and limited awareness of the disease [6]. The histological subtypes and immunohistochemical profiles of MBC are reported to be similar to those of postmenopausal FBC, with the notable exception of the rare occurrence of invasive lobular carcinoma [7].

Key risk factors for MBC include environmental influences, hormonal imbalances, and genetic mutations [8,9,10]. Notably, up to 4% of MBC patients carry a BRCA1 gene mutation, while 4% to 16% harbor a BRCA2 gene mutation [11].

Despite current guidelines recommending surgical approaches similar to those used for postmenopausal women [1], breast-conserving surgery (BCS) is still widely underutilized in MBC patients, with total mastectomy remaining the most commonly performed procedure [12,13].

The limited use of BCS may be partly attributed to low radiotherapy (RT) adherence rates reported in the literature [14,15] and the smaller breast tissue volume in men [16].

Although age- and stage-matched studies report similar overall survival (OS) and disease-free survival (DFS) rates between genders, MBC is generally associated with a poorer prognosis compared to FBC. This disparity is likely due to diagnostic delays, older age at diagnosis [17], and the generally shorter life expectancy of men [18].

The quality of life (QoL) among MBC patients has been poorly studied, with only limited available evidence. One study on tamoxifen-related side effects in MBC patients reported high hormone treatment discontinuation rates due to specific adverse effects, including weight gain, sexual dysfunction, and neurocognitive deficits [19]. A German study [20] assessing health-related QoL in men with BC found lower satisfaction and worse QoL compared to male reference populations. However, no study to date has specifically examined the impact of surgical treatment, particularly demolitive procedures, on QoL in MBC.

To address these significant gaps, we investigated the clinicopathological characteristics, therapeutic management, and follow-up of MBC patients who underwent surgery at our institution over a 20-year period. Notably, we developed a novel questionnaire to assess the QoL of men after BC surgery.

## 2. Materials and Methods

This single-institution retrospective cohort study was conducted at the Veneto Institute of Oncology (Padova, Italy) and adhered to the STROBE (Strengthening the Reporting of Observational Studies in Epidemiology) guidelines [21].

The study protocol was approved by the local ethics committee (CESC-IOV 2023-14).

### 2.1. Inclusion and Exclusion Criteria

The study included consecutive male patients with a new diagnosis of BC who underwent surgical treatment between January 1998 and December 2020. Clinicopathological data were sourced from our institutional database. Patients who underwent neoadjuvant treatments were included. Patients who underwent neoadjuvant treatments were also included. However, patients with metastatic disease, those lost to follow-up, or cases with incomplete clinical data were excluded.

### 2.2. Data Collection

Comprehensive clinical and tumor characteristic data were collected. Molecular subtypes were categorized according to the World Health Organization Classification of Tumors [22]. BC staging was performed following the eighth edition of the AJCC staging system [23]. Detailed records of surgical and medical treatments, including adjuvant RT, were meticulously compiled. Responses to therapy were assessed according to the RECIST criteria [24].

### 2.3. Study Endpoints

Survival outcomes, including OS and DFS, were analyzed, with recurrence categorized as loco-regional or distant. QoL and patients’ satisfaction with treatments and their outcomes were assessed using a novel 12-item scoring system based on a self-assessment questionnaire. The tool was divided into three distinct sections: satisfaction with surgery outcomes; perceived impact of medical treatments on daily life; and overall QoL. Each item was rated on a scale from 0 (worst outcome) to 3 (best outcome), resulting in section scores ranging from 0 to 12 and an overall score from 0 to 36. Global scores above 24 were considered indicative of good satisfaction or QoL (see Figure 6A for details). The questionnaire included six surgery-related questions (highlighted in red, Figure 6A) and six medical treatment-related questions (highlighted in blue, Figure 6A), allowing for a detailed sub-analysis of the specific impact of surgical versus medical treatments on QoL.

### 2.4. Statistical Analysis

Quantitative variables were described as median and interquartile ranges. Categorical variables were presented as counts and percentages. OS was defined as the time from diagnosis to death. DFS was calculated as the time from diagnosis to the occurrence of a documented disease progression, relapse, or death. Patients who did not experience an event during the study period were censored at the date of their last observation. Survival probabilities were estimated using the Kaplan–Meier method and compared among strata using the log-rank test. The 5-year survival probabilities were reported with their 95% confidence intervals (CIs). Hazard ratios (HRs) and their 95% CIs for each group were calculated using univariate Cox proportional hazard models. No deviations from the proportional hazard assumption were found by the Grambsch and Therneau statistical test. The independent influence of each covariate on survival was further analyzed in a multivariable Cox model, incorporating all characteristics significantly associated with outcomes in the univariate analyses.

The cumulative incidence function for each event type was estimated to provide the probability of experiencing death from breast cancer by a specific time, accounting for the competing risk of dying from other causes.

The scores from the different sections of the questionnaire were compared across different strata using the Kruskall–Wallis test. The comparison between the frequency of responses on a 0–3 scale to surgery-related and medical treatment-related questions was conducted using Chi-squared or Fisher’s exact test, as appropriate.

All statistical tests were two-sided, and a *p*-value < 0.05 was considered statistically significant. Statistical analyses were performed using the RStudio software version 4.4.2 (RStudio: Integrated Development Environment for R, RStudio Inc., Boston, MA, USA) [25].

## 3. Results

Over the 22-year study period, a total of 109 consecutive eligible male patients underwent surgical treatment for BC at our institution.

### 3.1. Patients’ Clinical Features

The clinical characteristics of all patients are detailed in Table 1A.

The median age of the entire cohort was 68 years (IQR 59–77; range 34–92), with all patients being Caucasians living in Italy. A family history of BC was observed in as many as 30.3% of patients, including two cases of MBC. Genetic testing was performed in 70.6% of patients, identifying genetic mutations in 23 cases (29.9% of those tested); specifically, the mutations involved the BRCA2 gene in 11 cases, the BRCA1 gene in 7 cases, and the PALB2 gene in 5 cases. Other recognized risk factors for MBC were observed at lower rates: 7.3% of the patients presented with gynecomastia, 3.7% had a history of cryptorchidism, and another 3.7% had occupational radiation exposure. Additionally, 28.4% of the cohort had a personal history of previous other malignancies, with the most common being prostate cancer (12.8%) and colorectal cancer (10.1%).

### 3.2. Clinical Presentation, Tumor Features, and Staging

All tumors were unifocal and unilateral, and most of them were located centrally (47.7%). The median tumor diameter was 21 mm (IQR 15–27 mm), and 80.7% of patients initially presented with a palpable mass. (Table 1B).

Ductal carcinoma in situ (DCIS) accounted for 7.3% of all cases, while the predominant histotype was an invasive BC of the non-special type, representing 89% of the cases. No cases of invasive lobular carcinoma were observed. MBCs were categorized as Luminal-like in nearly all cases (33% as Luminal A, 57.8% as Luminal B HER2-, and 4.6% as Luminal B HER2-positive, respectively), with one case of HER2-positive BC and four cases of triple-negative BC.

The majority of tumors were staged as T1 (48.6%) or T2 (40.4%). Lymph node metastases were detected in 52 patients (47.7%), with 23 being clinically node-positive at onset presentation. Overall, most patients were ultimately classified as stage I or II (34.9% and 40.4%, respectively).

### 3.3. Medical and Surgical Treatments

Two patients received neoadjuvant hormone therapy (HT), while nine underwent neoadjuvant chemotherapy (NACT), all achieving a partial clinical and radiological response to these treatments. (Table 1C).

Nearly all patients (96.3%) underwent total mastectomy, with only four cases of partial mastectomy. The sole sentinel lymph node biopsy (SLNB) was performed in 55 patients, while 23 underwent direct axillary lymph node dissection (ALND), and 29 underwent SLNB followed by ALND. Axillary surgery was omitted in two cases: one ductal carcinoma in situ (DCIS) with a tumor diameter under 1 cm, and one palliative procedure in a patient over 90 years old with significant comorbidities.

Overall, 56 postoperative complications were reported in 48 patients, with breast seroma being the most frequent (21 cases, 37.5% of all complications). The global lymphocele rate was 15.6%. It occurred in only 3 cases after SLNB (5.5%), while it was predominantly observed in patients who underwent ALND, with a relative rate of 26.9%. Four cases of postoperative bleeding required surgical re-intervention, while all other complications were managed conservatively.

Adjuvant chemotherapy (CT) was administered to 43 patients, while adjuvant HT was administered to 98 patients. Adjuvant radiotherapy (RT) was delivered to 47 patients: 4 following partial mastectomy, and 43 post-mastectomy chest wall RT, with 39 also receiving additional regional nodal RT.

### 3.4. Recurrences and Vital Status

Over a median follow-up time of 88 months (IQR 58–133), 19 patients (17.4%) died, with only 26.3% of these deaths caused by BC. The leading non-BC-related causes of death were cardiac events (36.8% of all deaths) and cancer at other sites (26.3%).

**Figure 1:**
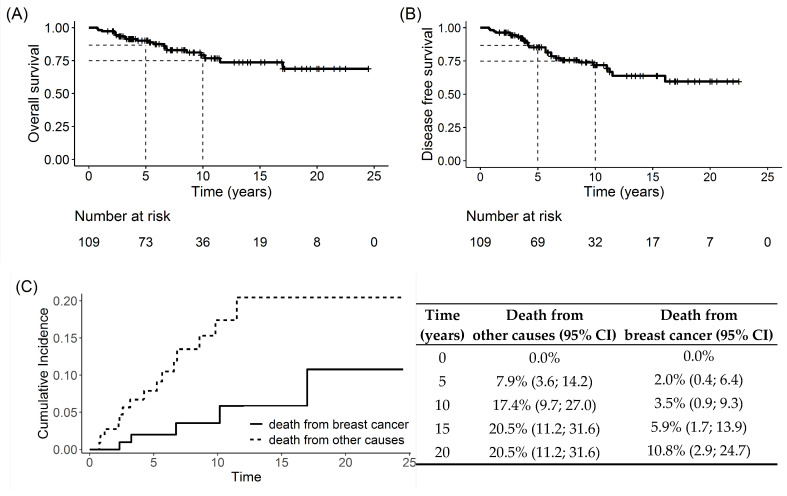
Overall survival (**A**) and disease-free survival (**B**) of the global population. Cumulative incidence rate of death from breast cancer versus other causes (**C**).

OS rates were 90.1% at 5 years and 79.1% at 10 years (Figure 1A). BC-specific mortality was 2% at 5 years and 3.5% at 10 years, while mortality due to other causes was 7.9% at 5 years and 17.4% at 10 years (Figure 1C).

DFS rates were 86.6% at 5 years and 74.8% at 10 years (Figure 1B).

Locoregional recurrences occurred in 11 patients, while 8 had distant metastases (4 in the lungs and 4 in the bones), and 3 patients experienced synchronous local and distant metastases (all of which had distant localizations in the bones). Locoregional recurrences were managed with surgery, RT, and/or CT (Table 1D).

Of the patients with distant metastases, 8 were treated with CT, whereas the remaining 3 received palliative care only.

**Figure 2:**
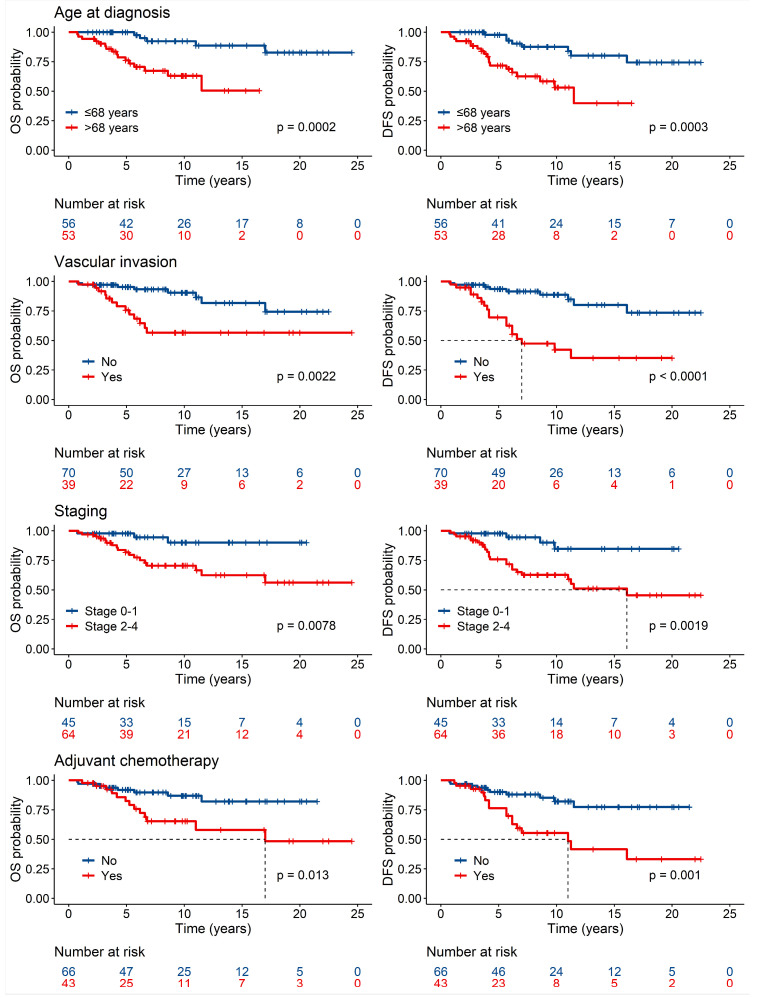
Overall survival and disease-free survival according to key significant factors.

Univariate Cox regression model analysis revealed that older age (*p* = 0.0002), undergoing ALND (*p* = 0.0008), the presence of vascular invasion (*p* = 0.0022), receiving adjuvant CT (*p* = 0.0126), and more advanced-stage diseases (*p* < 0.0001) [specifically, more advanced T-stage (*p* = 0.0285) and N-stage (*p* < 0.0001)] were significantly associated with poorer OS (Table 2A and Figure 2). Similarly, DFS was significantly impacted by older age (*p* = 0.0003), undergoing ALND (*p* < 0.0001), the presence of vascular invasion (*p* < 0.0001), more advanced-stage diseases (*p* < 0.0001) [specifically, more advanced T-stage (*p* = 0.0001) and N-stage (*p* < 0.0001)], and receiving adjuvant CT (*p* = 0.001). Additionally, receiving NACT (*p* = 0.0059) and higher tumor grading (*p* = 0.0171) were also associated with worse DFS outcomes (Table 2B and Figure 2).

**Figure 3:**
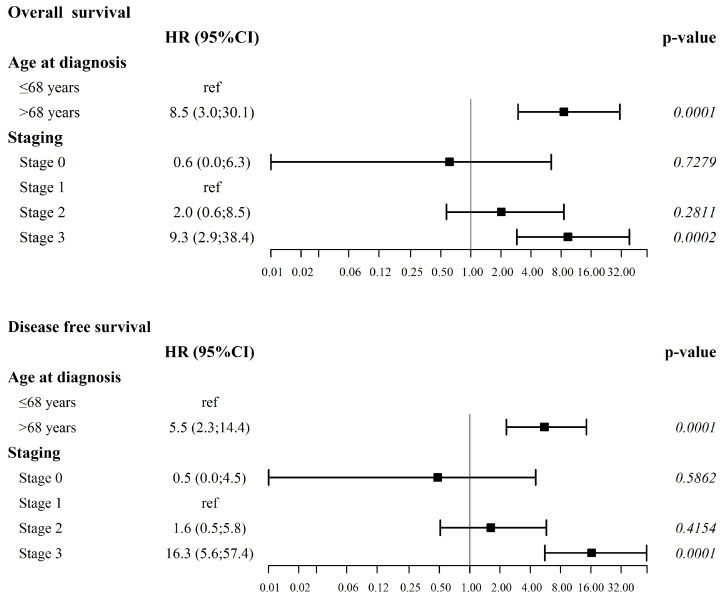
Multivariate forest plot analysis of the covariates significantly impacting overall survival and disease-free survival.

Multivariate analysis further delineated the independent roles of each selected covariate in predicting survival outcomes, revealing that older age at diagnosis (*p* < 0.0001 for OS and *p* = 0.0001 for DFS) and more advanced-stage diseases [with stage 3 associated with worse OS (*p* = 0.0002) and DFS, (*p* < 0.0001)] were the only independent factors significantly associated with worse outcomes for both OS and DFS (Figure 3).

### 3.5. Quality of Life

The QoL questionnaire was completed by 73 patients, representing 67% of the total cohort, with a median age of 65 years. All respondents had undergone surgical treatment and received at least one type of medical therapy. The median scores for each section (out of a maximum possible score of 12) were 9.0 for satisfaction with surgical treatments, 9.0 for satisfaction with medical treatments, and 10.0 for overall QoL. This resulted in a cumulative global median score of 28.5 (out of a maximum possible score of 36), with an interquartile range of 21.0–32.0.

**Figure 4:**
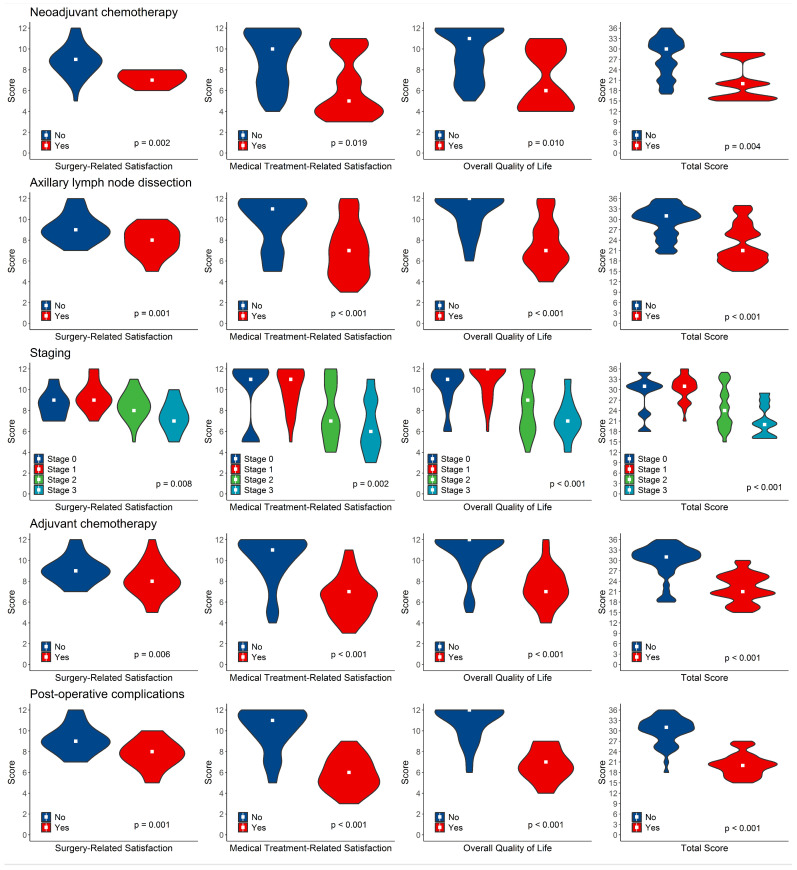
Impact of significant factors on quality of life and patients’ satisfaction scores: distribution and univariate analysis.

Univariate analysis identified several factors that significantly worsened all aspects of quality of life, and patient satisfaction was assessed through the questionnaire. Specifically, considering the global score derived from the sum of the scores across the three distinct sections, the following factors were associated with poorer QoL outcomes: receiving NACT (*p* = 0.004), receiving adjuvant CT (*p* < 0.001), undergoing ALND (*p* < 0.001), tumor staging (*p* < 0.001; with T-stage [*p* = 0.015] and N-stage [*p* < 0.001] significantly impacting QoL), and post-operative complications (*p* < 0.001) (Figure 4).

**Figure 5:**
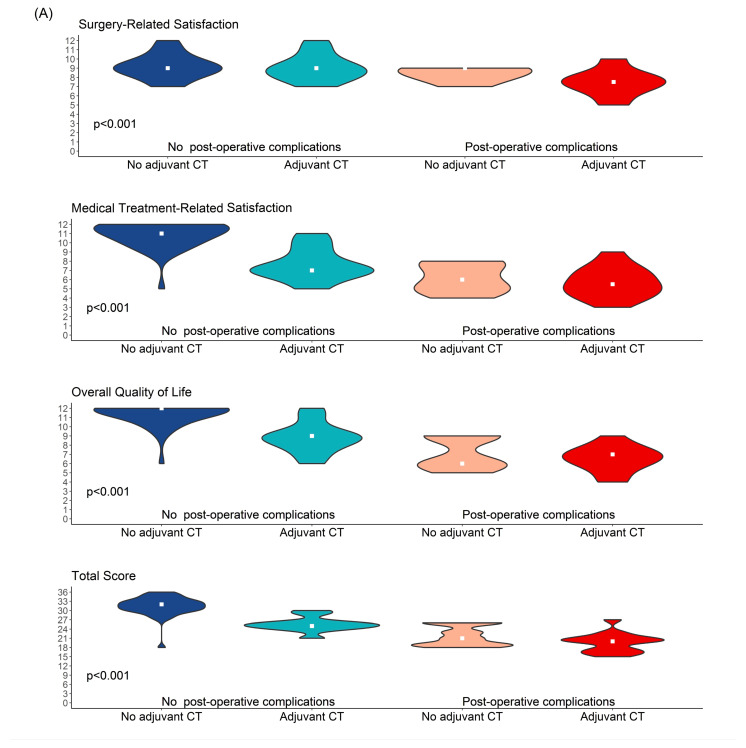

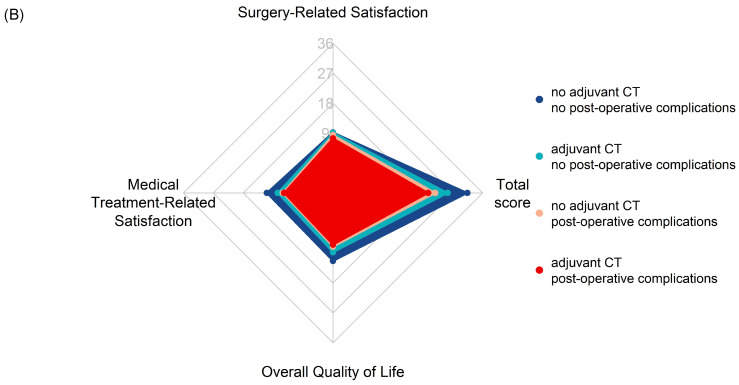
Violin (**A**) and radar (**B**) plots depicting multivariate analysis of quality of life and patient satisfaction scores.

Multivariate analyses revealed that receiving adjuvant CT was an independent factor significantly associated with worse outcomes in medical treatment-related satisfaction scores (*p* < 0.001) and overall QoL scores (*p* < 0.001). Similarly, post-operative complications were identified as an independent factor significantly associated with worse outcomes in both surgery-related satisfaction scores and overall QoL scores (*p* < 0.001 for both) (Figure 5).

**Figure 6:**
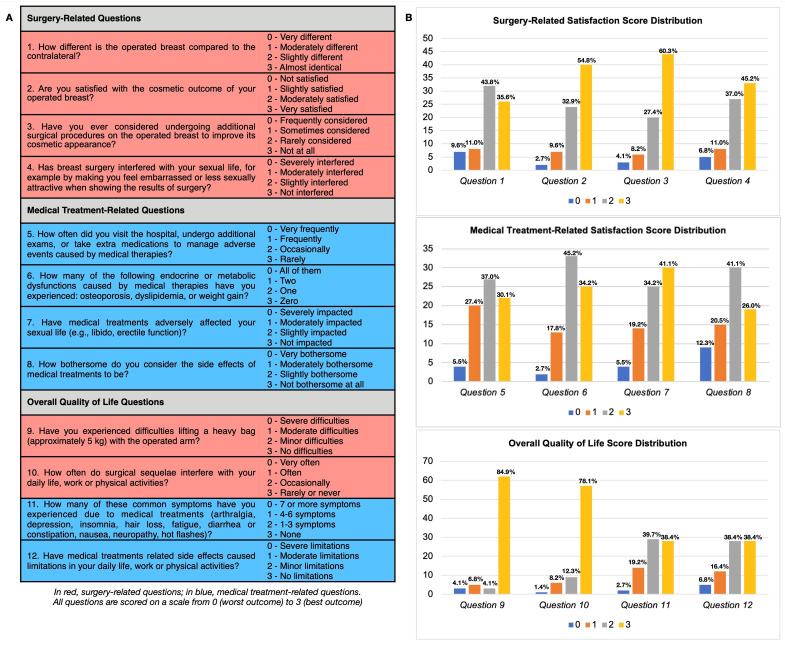
Survey questions administered to patients (**A**) and distribution of patient response scores (**B**).

As shown in Figure 6, surgery (which, in nearly all cases, involved total mastectomy with the removal of the nipple–areola complex) had a minimal impact on aesthetic satisfaction and sexual life in MBC patients. The majority (79.5%) of respondents reported a score of 2 or 3 (out of 3) for question 1, indicating that they perceived the operated breast as similar to or only slightly different from the contralateral breast. Similarly, 87.7% of respondents scored 2 or 3 (out of 3) for question 2, expressing satisfaction with the cosmesis of the operated breast. Additionally, 82.2% scored 2 or 3 (out of 3) for question 4, highlighting that breast surgery outcomes had a low impact on the sexual life of MBC patients.

Conversely, medical therapies had a significant negative impact on QoL and the sexual life of MBC patients. A notable 24.7% of respondents reported decreased libido or erectile dysfunction, scoring 0 or 1 (out of 3) on question 7. The majority of respondents (67.1%) scored 2 or 3 (out of 3) for question 8, highlighting that the side effects of medical treatments were perceived as significantly bothersome. Additionally, a substantial proportion of patients (69.9%) scored 0, 1, or 2 on question 5, indicating that they were frequently forced to seek additional care or treatments to manage these adverse effects.

Surgery did not appear to cause significant limitations in daily life activities, with 90.4% of respondents reporting a low impact of surgical sequelae (scoring 2 or 3 on question 10) and 84.9% of respondents experiencing no difficulties lifting a heavy bag (score 3 on question 9). Conversely, side effects related to medical therapies were frequent: 16 patients experienced more than four concurrent side effects (scoring 0 or 1 on question 11), and 23.2% of respondents rated these side effects as causing severe or moderate limitations in daily life activities (scoring 0 or 1 on question 12).

The sub-analysis comparing the frequency distribution of responses to surgery- and medical treatment-related questions revealed that patients reported a higher frequency of the best score for surgery-related items with respect to medical treatment-related items (mean percentage of 59.8% versus 34.7%, *p* < 0.001). This difference highlights that medical therapies had a greater adverse impact on patients’ satisfaction and QoL compared to surgery.

## 4. Discussion

This large monocentric retrospective cohort study provides a comprehensive analysis of MBC clinical–pathological features, treatments, and follow-up over 2 decades. It represents one of the largest single-institution series published to date, according to our knowledge. Compared to female populations, invasive lobular carcinoma is notably underrepresented in males, likely due to anatomical differences and the lack of terminal lobules in the male breast [26]. This is consistent with our findings.

Staging emerged as the primary determinant of OS and DFS, with histological lymph node status playing a crucial role in prognosis. Additionally, age, vascular invasion, and receiving CT were significantly associated with worse survival and higher recurrence rates.

QoL of MBC patients and their satisfaction with the treatments received have been underinvestigated to date. To the best of our knowledge, this is the first study to systematically and thoroughly evaluate these aspects, particularly focusing on the different impacts of surgical and medical treatments on patients’ QoL. Despite the predominance of mastectomies in our cohort, male patients reported high levels of post-surgical satisfaction. The main factors influencing the overall QoL score were receiving adjuvant chemotherapy and post-operative complications, with the latter being the only factor significantly impacting satisfaction with aesthetic outcomes. Medical therapies appeared to have a greater negative impact on QoL compared to surgery, as demonstrated by a dedicated analysis of specific questions. Over the span of this study, no significant changes in the surgical approach were observed, with BCS performed only in a limited number of selected cases. The predominance of mastectomy aligns with local guidelines but may also reflect male patients’ preferences and heightened concerns about recurrence risks over cosmetic outcomes. Conversely, in females, the breast is considered a significant marker of femininity and maternity, and advances in oncological and technical fields have significantly reduced mastectomy rates in favor of BCS in women. This underscores the need for further research and consideration of patient-specific factors to optimize surgical decision-making in MBC cases.

### 4.1. MBC Histopathological Characteristics, Prognostic Factors, and Survival Outcomes

Our findings align with major studies in the field. Among the cohort, 89% presented with invasive carcinoma of no special type, mirroring data from the literature, which confirms this as the most common histological subtype in both male and FBC populations [7]. The low incidence of DCIS (only 7.3% of the diagnoses in our population) may be partially attributed to the absence of screening programs for men. A landmark analysis of 1500 MBC cases demonstrated that 99% of tumors were estrogen receptor (ER)-positive, 81% were progesterone receptor (PR)-positive, and 97% were androgen receptor (AR)-positive [7]. Similarly, 95% of invasive MBCs in our cohort were ER-positive, reinforcing the value of hormone therapy. AR positivity in MBC has garnered increasing attention due to its potential as a therapeutic target. Emerging therapies, such as AR inhibitors, are being explored, particularly for ER-negative/AR-positive tumors, representing a rare but potentially actionable subgroup. The predominance of ER-positive tumors underscores the importance of endocrine therapies in MBC treatment. Aromatase inhibitors, tamoxifen, and GnRH analogues remain the cornerstone of therapy for hormone receptor-positive disease. However, studies have noted differences in treatment adherence and side effect profiles in male patients compared to females, warranting tailored approaches to optimize outcomes [18]. According to the literature [27], hormone receptor status has been shown to significantly influence survival, with ER-negative tumors associated with worse outcomes. Donegan et al. [28] reported a 5-year OS rate of 78% for ER-positive patients compared to 25% for ER-negative patients. However, in our series, the limited number of ER-negative cases makes direct comparisons challenging. Among these, only one death was recorded, and it was not related to BC. Triple-negative BC was rare in our cohort, accounting for 3.7% of cases, which aligns with the even lower incidence of 0.3% reported by Cardoso et al. [7].

DFS and OS were strongly influenced by lymph node status, a well-established and significant prognostic indicator in concordance with several other studies highlighting the dramatic impact of lymph node involvement on survival outcomes. For instance, Guinee et al. [29] reported 10-year DFS rates of 84%, 44%, and 14% for pN0, pN1–3, and pN > 3 groups, respectively, in a cohort of 224 men undergoing axillary dissection. Similarly, Borgen et al. [30] identified axillary lymph node status as the most powerful predictor of survival outcomes, with 10-year OS rates of 80% for node-negative (pN0) patients and 35% for node-positive (pN+) patients. Our data corroborate this, showing a 10-year OS of 82.4% for pN0 patients versus 48% for pN+ patients.

MBC is typically diagnosed at an older age compared to FBC [31], particularly in regions where routine mammographic screening for women is widespread [32]. In our study, the median age at diagnosis was 68 years, which is slightly higher than the 62.6 years reported in a systematic review by De La Cruz et al. [31]. Advanced age at diagnosis emerged as a significant determinant of worse OS and DFS. The lower awareness among the male population, combined with the absence of routine screening programs, contributes to delayed diagnoses, often occurring only when the disease becomes palpable. This is reflected by our findings, where 80.7% of patients presented with a palpable mass. Furthermore, axillary involvement at diagnosis is more common in MBC than in FBC cohorts [5]. This is further confirmed by our study, with 47.7% of patients presenting with pathological lymph node involvement at the time of diagnosis, suggesting more aggressive or advanced disease at presentation. These findings align with contemporary research highlighting disparities in awareness and diagnostic practices between men and women. For example, studies have suggested that public health campaigns targeting male populations could help bridge this gap by emphasizing the importance of early detection and self-awareness of breast abnormalities [18].

Despite advancements in screening and treatment strategies that have significantly reduced women’s BC mortality, the prognosis for MBC has not improved over the past 3 decades [33]. Greif et al. [34] compared 13,457 MBC cases to 1,439,866 BC cases in women, reporting that men presented with larger tumors (median size 20 mm vs. 15 mm in women, compared to 21 mm in our cohort) and were less likely to have low-grade tumors (16.0% vs. 20.7% in women, and 9.2% in our cohort) compared to women. Additionally, men were more likely to have lymph node metastases (41.9% vs. 33.2% in women and 47.7% in our study). Mastectomy rates were significantly higher among men compared to women (67% vs. 38% in Greif’s study and 96.3% in our cohort). While some studies, such as those by Madden et al. [35] and Ribeiro et al. [36], have shown no significant differences in OS between BCS and mastectomy, O’Malley et al. [37] observed worse OS for both Caucasian and Black male patients undergoing BCS compared to those receiving mastectomies. However, a systematic review of MBC cohort studies by Sauder et al. [12] reported equivalent 5-year disease-specific survival and global OS for mastectomy and BCS. These findings suggest that BCS could be a viable and equally effective treatment option for MBC. However, concerns remain due to low rates of RT compliance (possibly due to logistical barriers and a lack of awareness regarding its benefits) and the high preference for mastectomy observed among male patients. This highlights the importance of shared decision-making in selecting the most appropriate treatment strategy for MBC patients, which has been shown to improve treatment satisfaction and adherence [38]. In our cohort, post-operative complication rates following mastectomies in MBC patients were comparable to those observed in female populations treated with BCS [39]. This is likely attributable to the smaller volume of breast tissue and skin excised during demolitive surgical procedures in men. Future studies could further investigate strategies to optimize surgical approaches while better preserving quality-of-life and aesthetic outcomes in MBC patients.

### 4.2. Quality of Life in MBC

QoL assessments specifically tailored to MBC remain notably limited in the literature, probably due to the rarity of the disease. However, insights from the International Male Breast Cancer Program [40] suggest that QoL in MBC patients may be comparable, if not superior, to that observed in FBC patients. This aligns with findings from a comprehensive German survey conducted between 2006 and 2010 comparing 84 men treated for BC with 20,589 women. The study revealed that men had significantly better scores in physical functions, bodily pain, role functioning, and mental health [20]. Nevertheless, when compared to the general healthy male population, men with BC reported poorer emotional and physical functioning scores [20].

In contrast, Avila et al. [41] reported several late effects from BC treatments in men, including lymphedema, impaired arm and shoulder movement, hair loss, and sexual dysfunction. Moreover, Schroder et al. noted that sexual activity in men is more severely affected by advanced disease stages and older age at diagnosis [40]. The psychological impact of MBC is also significant. A metasynthesis of qualitative studies highlighted that men often experience feelings of isolation and stigma due to the perception of BC as a female disease. This can lead to delays in seeking treatment and a reluctance to discuss their condition openly [42]. These findings underline the multifaceted challenges faced by male patients, particularly in managing long-term treatment effects.

Interestingly, men appear less affected by the aesthetic outcomes of demolitive breast surgery, likely due to reduced societal emphasis on male breast appearance and less importance of its role in sexual life. Nevertheless, advancements in surgical management could offer new opportunities for enhancing QoL, such as the adoption of nipple-sparing mastectomy (NSM), when oncologically feasible and safe. MBCs are often located in the retroareolar region, making nipple–areola complex preservation oncologically challenging in many cases. However, in routine clinical practice, mastectomy with nipple–areola complex removal is often performed even when the tumor is located at a distance from the nipple–areola complex, which could potentially be preserved. NSM has already demonstrated significant benefits in female populations, including improved body image and satisfaction, without compromising oncological safety in carefully selected cases [7]. In male patients, preserving the nipple–areola complex could substantially enhance post-surgical satisfaction by maintaining a more natural chest contour. Due to the less extensive breast tissue in men, NSM might also be technically easier to perform and could be incorporated into surgical practice with careful patient selection and robust oncological protocols.

Hormonal side effects from medical treatments significantly impair sexual performance in male patients [43]. HT-related side effects, such as erectile dysfunction, a decrease in libido, and hair loss, differ from those typically observed in female populations, and this should be thoroughly discussed with patients to manage expectations and improve overall treatment satisfaction [44]. Emerging evidence suggests that tailored hormonal management, potentially integrating therapies to mitigate adverse effects, could enhance QoL outcomes for male patients.

Our QoL and patient satisfaction questionnaire clearly demonstrated that MBC patients were generally satisfied with the outcomes of treatments received, with a median global score of 28.5 out of 36. However, a deeper analysis and comparison of scores related to surgical outcomes versus those related to medical treatment outcomes revealed that surgical treatments had a significantly lower impact on overall QoL and patient satisfaction compared to medical therapies.

Compared to women with BC, male patients frequently lack social support and are less inclined to join patient associations [45,46]. Furthermore, a recent qualitative study emphasized that men diagnosed with BC face unique challenges, including a lack of tailored information and support services. Participants expressed a desire for more resources, specifically addressing MBC to help navigate their diagnosis and treatment [47]. These findings underscore the importance of developing supportive care strategies that address the unique needs of MBC patients. Healthcare providers should be aware of the potential psychological and social challenges faced by this population and strive to offer comprehensive support that encompasses both physical and emotional well-being.

### 4.3. Study Limitations

The retrospective design and demographic uniformity of our cohort, composed exclusively of Caucasian patients, may limit the generalizability of our findings. The inherently small sample size, a common limitation in MBC studies due to the rarity of the disease, constrains our ability to draw definitive conclusions. This limitation also hampers the robustness of statistical analyses, particularly when comparing subgroups. Additionally, there is a notable discrepancy in clinical practices between our institution and other BC centers [34], as BCS was rarely performed in our cohort. This divergence may reflect institutional preferences, perceived risks and benefits, or patient-specific factors and preferences, potentially limiting the applicability of our findings to settings where BCS is more commonly employed. The extended observation period of this study is another limitation, as it may introduce variability in treatment approaches over time, particularly regarding the indications for medical therapies. However, consistent with findings reported in other recently published studies [48], no significant changes in surgical trends were observed, with mastectomy remaining the preferred treatment method throughout the study period. The QoL questionnaire used in this study, while novel and specifically designed to investigate QoL in MBC survivors, was not externally validated. Although its simplicity and effectiveness contributed to high response rates, the lack of external validation limits its comparability with other widely used QoL assessment tools. This underscores a significant gap in validated QoL instruments tailored specifically for men with BC, which warrants further development and standardization. Additionally, while our study provides valuable insights into the impact of surgical and medical treatments on QoL, it does not fully account for potential psychological and emotional dimensions, such as stigma and isolation, which are known to affect men with BC. These aspects could be explored in future studies through qualitative research or mixed-method approaches [49]. Despite these limitations, our study provides valuable contributions to the understanding of MBC, particularly in QoL outcomes. It highlights the unique challenges of managing this rare condition and provides a foundation for future research aimed at improving clinical practices and patient-centered care.

## 5. Conclusions

MBC remains a rare condition, yet its incidence is increasing globally. There is a critical need to raise awareness among both patients and clinicians, particularly within high-risk populations. Due to the lack of clinical trials specifically designed for male patients, treatment strategies for MBC often rely on recommendations derived from studies conducted on FBC patients. However, the distinct clinical–pathological features of MBC highlight the pressing need for prospective studies and clinical trials tailored to this demographic.

While larger, multicenter studies involving more patients and comparisons with healthy individuals and FBC patients are essential, single-institution studies like ours continue to provide valuable insights into this underexplored population.

The study highlights the need for shared decision-making to ensure that male patients can make informed choices about their surgical options, potentially integrating innovative approaches such as nipple-sparing mastectomy. Such techniques, where oncologically feasible, could significantly improve post-surgical satisfaction and overall QoL.

QoL in MBC remains understudied. This study offers unique insights that are crucial for understanding patient needs and guiding treatment strategies. Adjuvant chemotherapy was identified as the single-most significant factor negatively impacting QoL. Notably, QoL impairments were more strongly associated with the side effects of medical therapies, such as chemotherapy and HT, than with the consequences of surgery. This finding contrasts with observations in FBC survivors, where cosmetic outcomes often play a larger role in QoL considerations, and is also likely due to reduced concern about cosmetic outcomes in the male population.

While MBC patients may experience QoL outcomes comparable to or even better than FBC patients in certain domains, specific psychological and social challenges require targeted interventions. Future research should prioritize clinical trials tailored to MBC patients, patient-centered care strategies, and validated QoL tools to address these unmet needs and improve both survival and QoL outcomes.

## Figures and Tables

**Table 1:** (**A**) Family and medical history of the study population. (**B**) Clinico-pathological characteristics of study population. (**C**) Treatment approaches of the study population. (**D**) Oncological outcomes of the study population.

		Overall Population (N = 109)
(**A**)		
**Age (years)**	Median (IQR)	68 (59–77)
	Range	34–92
**Family history of BC**	No	76 (69.7)
	Yes	33 (30.3)
**Genetic test**	Not performed	32 (29.4)
	Performed	77 (70.6)
**Genetic mutations detected**	No	54/77 (70.1)
	Yes	23/77 (29.9)
	BRCA2 mutation	11/23 (47.8)
	BRCA1 mutation	7/23 (30.4)
	PALB2 mutation	5/23 (21.7)
**Risk factors**	Gynecomastia	8 (7.3)
	Radiation exposure	4 (3.7)
	Testicular abnormalities	4 (3.7)
	Klinefelter syndrome	1 (0.9)
	Cirrhosis	1 (0.9)
**Previous neoplasm**	Prostate	14 (12.8)
	Colon	11 (10.1)
	Lung	3 (2.8)
	Melanoma	2 (1.8)
	Pancreas	1 (0.9)
(**B**)		
**Tumor macroscopic** **features**	Unilateral	109 (100)
	Unifocal	109 (100)
**Site**	Central/Retroareolar	78 (71.6)
	Upper-outer quadrant	11 (10)
	Upper-inner quadrant	8 (7.3)
	Lower-outer quadrant	7 (6.4)
	Lower-inner quadrant	5 (4.6)
**Size (mm)**	Median (IQR)	21 (15–27)
**First clinical symptom**	Palpable mass	88 (80.7)
	Nipple/skin changes	34 (31.2)
	Mastodynia	4 (3.7)
**Histotype**	Invasive carcinoma of non-special type	97 (89.0)
	Ductal carcinoma in situ	8 (7.3)
	Papillary	2 (1.8)
	Cribriform	1 (0.9)
	Cystic adenoid	1 (0.9)
**Grading**	G1	10 (9.2)
	G2	50 (45.9)
	G3	49 (45.0)
**Vascular invasion**	Absent	70 (64.2)
	Present	39 (35.8)
**Luminal classification**	Luminal A	36 (33.0)
	Luminal B (Ki67 ≥ 20%)	63 (57.8)
	Luminal B (HER2 positive)	5 (4.6)
	HER2 positive	1 (0.9)
	Triple negative	4 (3.7)
**pT**	IS	8 (7.3)
	1	53 (48.6)
	2	44 (40.4)
	3	0 (0.0)
	4	4 (3.7)
**cN**	cN0	86 (78.9)
	cN+	23 (21.1)
**pN**	0	55 (50.5)
	1	37 (33.9)
	2	11 (10.1)
	3	4 (3.7)
	X	2 (1.8)
**Stage**	IS	8 (7.3)
	I	38 (34.9)
	II	44 (40.4)
	III	19 (17.4)
(**C**)		
**Neoadjuvant** **Hormonotherapy**	Administered	2 (1.8)
	Median duration [months (IQR)]	14.5 (9.75–19.25)
**Neoadjuvant** **Chemotherapy**	Administered	9 (8.3)
	Median duration [months (IQR)]	5.0 (5.0–6.0)
**Breast surgery**	Total mastectomy	105 (96.3)
	Partial mastectomy	4 (3.7)
**Axillary surgery**	Sentinel lymph node biopsy	55 (50.5)
	Axillary lymph node dissection	23 (21.1)
	SLNB + ALND	29 (26.6)
	Not performed	2 (1.8)
**Complications**	Seroma	21 (19.3)
	Lymphocele	17 (15.6)
	Bleeding	7 (6.4)
	Lymphedema	6 (5.5)
	Wound infection	3 (2.8)
	Flap ischemia	2 (1.8)
**Re-intervention**		4 (3.7)
**Adjuvant** **Hormonotherapy**	Administered	98 (89.9)
	Median duration [months (IQR)]	56.8 (51.2–59.4)
**Adjuvant** **Chemotherapy**	Administered	43 (39.4)
	Median duration [months (IQR)]	7.3 (4.5–11.7)
**Radiotherapy**	Administered	47 (43.1)
	Whole breast RT after partial mastectomy	4/47 (8.5)
	Chest wall RT after mastectomy	43/47 (91.5)
	Regional nodal irradiation	39/47 (83.0)
(**D**)		
**Recurrences**	Total	22 (20.2)
	Loco-regional	11/22 (50.0)
	Distant metastases	8/22 (36.4)
	Synchronous loco-regional and distant metastases	3/22 (13.6)
**Metastases sites**	Bone metastases	7/11 (63.6)
	Lung metastases	4/11 (36.4)
**Recurrence treatment**	Chemotherapy	13/22 (59.1)
	Surgery	9/22 (40.9)
	Palliative cares	3/22 (13.6)
	Radiotherapy	2/22 (9.1)
**Vital status**	Alive	90 (82.6)
	Dead	19 (17.4)
	Death due to BC	5/19 (26.3)
	Death due to no-BC causes	14/19 (73.7)
	Cardiac events	7/19 (36.8)
	Other neoplasms	5/19 (26.3)
	Pneumonia	1/19 (5.3)
	Diabetes mellitus	1/19 (5.3)

BRCA indicates breast cancer gene; PALB, partner and localizer of BRCA; IQR, interquartile range; G1, well differentiated; G2, moderately differentiated; G3, poorly differentiated; SLNB, sentinel lymph node biopsy; ALND, axillary lymph node dissection; RT, radiotherapy; BC, breast cancer. Numbers are frequencies and percentages.

**Table 2:** (**A**) Univariate analyses evaluating the impact of each factor on overall survival. (**B**) Univariate analyses evaluating the impact of each factor on disease-free survival.

		E/N	5-Year OS (95% CI)	logrank *p*	HR (95% CI)	*p*-Value
(**A**)						
**Age at diagnosis**	≤68 years	5/56	100% (-)	**0.0002**	ref	
	>68 years	16/53	76.2% (60.9; 86.1)		5.86 (2.19; 19.28)	**0.0002**
**NACT**	No	17/98	90.8% (82.3; 95.3)	0.1190	ref	
	Yes	4/11	70.1% (32.3; 89.5)		2.54 (0.79; 6.6)	0.1103
**ALND**	No	4/57	95.9% (84.4; 99)	**0.0008**	ref	
	Yes	17/52	80.5% (65.7; 89.4)		4.88 (1.87; 15.8)	**0.0008**
**Vascular invasion**	Absent	8/70	95.3% (86; 98.5)	**0.0022**	ref	
	Present	13/39	75.6% (57; 87.1)		3.54 (1.52; 8.73)	**0.0034**
**Grading**	G1	0/10	100% (-)	0.0683	ref	
	G2	7/50	94.8% (80.7; 98.7)		3.36 (0.41; 436.48)	0.3199
	G3	14/49	79.6% (64.4; 88.9)		6.81 (0.91; 870.18)	0.0656
**Luminal type**	Luminal A	6/36	93.6% (76.6; 98.4)	0.7735	ref	
	Luminal B	14/68	84.7% (72.5; 91.8)		1.41 (0.58; 3.83)	0.4627
	HER2 positive	0/1	100% (100; 100)		1.52 (0.01; 13.17)	0.7895
	Triple negative	1/4	100% (100; 100)		2.42 (0.25; 11.76)	0.3769
**pT-TNM**	TIS	0/8	100% (-)	**0.0285**	ref	
	T1	7/53	93.6% (81.2; 97.9)		2.25 (0.27; 292.1)	0.5309
	T2	12/44	84% (67.5; 92.6)		4.92 (0.65; 631.01)	0.1515
	T4	2/4	33.3% (0.9; 77.4)		16.15 (1.3; 2236.33)	**0.0298**
**pN-TNM**	N0	7/57	92.4% (80.8; 97.1)	**<0.0001**	ref	
	N1	6/37	94.3% (78.9; 98.5)		1.32 (0.44; 3.83)	0.6044
	N2	4/11	77.1% (34.5; 93.9)		4.08 (1.15; 12.92)	**0.0315**
	N3	4/4	25% (0.9; 66.5)		15.84 (4.34; 52.21)	**0.0002**
**Stage**	IS	0/8	100% (-)	**<0.0001**	0.66 (0; 6.83)	0.7743
	I	3/38	97.3% (82.3; 99.6)		ref	
	II	6/44	94.6% (79.9; 98.7)		1.5 (0.42; 6.3)	0.5362
	III	9/19	59.3% (30.5; 79.5)		7.81 (2.46; 31.42)	**0.0004**
**Adjuvant chemotherapy**	No	8/66	91.9% (81.6; 96.6)	**0.0126**	ref	**<0.0001**
	Yes	13/43	82.4% (64.6; 91.8)		2.85 (1.23; 7.03)	**0.0150**
**Genetic mutation**	No	8/54	91.2% (78.1; 96.6)	0.3817	ref	
	Yes	2/23	94.7% (68.1; 99.2)		0.6 (0.11; 2.17)	0.4547
(**B**)						
**Age at diagnosis**	≤68 years	8/56	97.8% (85.3; 99.7)	**0.0003**	ref	
	>68 years	19/53	71.8% (56.1; 82.7)		4.2 (1.87; 10.33)	**0.0004**
**NACT**	No	21/98	88.3% (79.2; 93.6)	**0.0059**	ref	
	Yes	6/11	58.4% (22.7; 82.3)		3.54 (1.34; 8.14)	**0.0131**
**ALND**	No	4/57	95.9% (84.4; 99)	**<0.0001**	ref	
	Yes	23/52	73.6% (58.1; 84.2)		7.48 (2.96; 23.83)	**<0.0001**
**Vascular invasion**	Absent	9/70	93.6% (83.7; 97.6)	**<0.0001**	ref	
	Present	18/39	69.6% (50.6; 82.4)		4.79 (2.23; 10.99)	**0.0001**
**Grading**	G1	0/10	100% (-)	**0.0171**	ref	
	G2	9/50	92.2% (77.6; 97.4)		4.23 (0.54; 545.07)	0.2132
	G3	18/49	75% (59.2; 85.4)		9.45 (1.29; 1203.04)	**0.0204**
**Luminal type**	Luminal A	7/36	90.3% (72.8; 96.8)	0.3869	ref	
	Luminal B	18/68	82.9% (70.3; 90.5)		1.52 (0.67; 3.8)	0.3208
	HER2 positive	1/1	100% (100; 100)		7.2 (0.75; 33.68)	0.0779
	Triple negative	1/4	75% (12.8; 96.1)		2.3 (0.24; 10.68)	0.3994
**pT-TNM**	TIS	0/8	100% (-)	**0.0001**	0.34 (0; 2.65)	0.3746
	T1	9/53	89.3% (76.1; 95.4)		ref	
	T2	15/44	81.2% (64.2; 90.6)		2.17 (0.98; 5.05)	0.0555
	T4	3/4	33.3% (0.9; 77.4)		12.33 (3; 40.61)	**0.0016**
**pN-TNM**	N0	8/57	92.4% (80.8; 97.1)	**<0.0001**	ref	
	N1	8/37	90.6% (73.4; 96.9)		1.55 (0.59; 4.08)	0.3706
	N2	7/11	53.9% (17.6; 80.2)		8.95 (3.17; 24.89)	**0.0001**
	N3	4/4	25% (0.9; 66.5)		18.76 (5.08; 62.53)	**0.0001**
**Stage**	IS	0/8	100% (-)	**<0.0001**	0.5 (0; 4.64)	0.6048
	I	4/38	97.3% (82.3; 99.6)		ref	
	II	7/44	91.6% (75.9; 97.2)		1.26 (0.4; 4.46)	0.6993
	III	13/19	45.4% (19.4; 68.3)		14.22 (5.02; 48.7)	**<0.0001**
**Adjuvant chemotherapy**	No	10/66	90.1% (79.2; 95.4)	**0.0010**	ref	
	Yes	17/43	76.4% (57.9; 87.6)		3.36 (1.58; 7.5)	**0.0016**
**Genetic mutation**	No	11/54	86.9% (72.9; 93.9)	0.5949	ref	
	Yes	4/23	90% (65.3; 97.4)		0.79 (0.24; 2.22)	0.6673

E/N, events/total number of patients; DFS, disease-free survival; NACT, neoadjuvant chemotherapy; ALND, axillary lymph nodes dissection; CI, confidence interval; HR, hazard ratio; ref, reference; OS, overall survival. All significant *p* values (<0.05) are highlighted by bold characters.

## Data Availability

The datasets generated and analyzed during the current study are not publicly available due to our institution’s policies and the need to protect study participants’ privacy. However, they are partially available from the corresponding author upon reasonable request.
